# Potential value of serum brain-derived neurotrophic factor, vascular endothelial growth factor, and S100B for identifying major depressive disorder in knee osteoarthritis patients

**DOI:** 10.3389/fpsyt.2022.1019367

**Published:** 2022-10-25

**Authors:** Peng Zhang, Yuyuan Xiong, Bangjun Wang, Yi Zhou, Zijian Wang, Jiaqi Shi, Chao Li, Xinyan Lu, Gang Chen

**Affiliations:** ^1^Department of Orthopedics, Xiangyang Central Hospital, Affiliated Hospital of Hubei University of Arts and Science, Xiangyang, Hubei, China; ^2^Department of Thyroid and Breast Surgery, Xiangyang Central Hospital, Affiliated Hospital of Hubei University of Arts and Science, Xiangyang, Hubei, China

**Keywords:** osteoarthritis, major depression disorder, brain-derived neurotrophic factor, vascular endothelial growth factor, S100B, follow-up

## Abstract

**Background:**

The chronic pain and functional limitations in osteoarthritis (OA) patients can increase risk of psychiatric disorders, e.g., major depression disorder (MDD), which may further aggravate the clinical symptoms of OA. Early detection of MDD is essential in the clinical practice of OA.

**Materials and methods:**

Two hundred and fifteen participants with knee OA were recruited, including 134 MDD patients (i.e., MDD group) and 81 ones without MDD (i.e., control group). Among them, 81 OA participants in the control group received a 3-year follow-up and were divided into trans-MDD group (who transforming into MDD; *N* = 39) and non-MDD group (who keeping non-MDD; *N* = 42) at the end of the follow-up. The 17-item Hamilton Depression Scale (HAMD-17), Self-Rating Depression Scale (SDS), and Visual Analogue Scale (VAS) were performed. Furthermore, serum levels of brain-derived neurotrophic factor (BDNF), vascular endothelial growth factor (VEGF), S100B, and IGF-1 were detected.

**Results:**

(1) Compared with OA participants without MDD, there were significant decrease in serum BDNF and significant increase in serum VEGF and S100B and VAS scores in OA participants with MDD. (2) A mediation of the association was found between the VAS scores and the HAMD-17 scores through the BDNF as mediator in OA participants with MDD. (3) Significantly lower baseline BDNF levels and higher baseline S100B levels were detected in OA participants who transforming to MDD after a 3-year follow-up when compared with those who keeping non-MDD. (4) In the trans-MDD group, significant associations of the change of serum BDNF levels with rate of change of HAMD-17 scores were found, and baseline serum S100B levels positively correlated with the HAMD-17 scores at the end of the follow-up. (5) In OA participants, the composite indicator of BDNF, VEGF, and S100B differentiated MDD patients from controls with the area under the curve (AUC) value of 0.806, and the combined indicator of baseline BDNF and S100B distinguished trans-MDD participants from non-MDD ones with an AUC value of 0.806.

**Conclusion:**

Serum BDNF, VEGF, and S100B may be potential biomarkers to identify MDD in OA patients. Meanwhile, serum BDNF and S100B shows great potential to predict the risk of MDD for OA.

## Introduction

Osteoarthritis (OA) is a common cause of disability and source of societal cost in elders, with 250 million persons being currently affected in the world (1). As a whole joint disease, the complex pathogenesis of OA involving in mechanical, inflammatory, and metabolic factors, can induce structural destruction of the synovial joint (2). Pain is the dominant symptom of OA, and in particular, the pain in knee OA is considered as typically transitioning from intermittent weight-bearing pain to a chronic pain (3). Due to the current clinical therapies are difficult to cure OA completely, the chronic pain and functional limitations can increase risk of negative psychological outcomes, such as major depression disorder (MDD), which will further diminishes quality of life for OA patients (4–6). Furthermore, individuals with MDD exhibit more significant anhedonia and greater daily negative affect compared to non-depressed ones (7), which may aggravate the feelings of pain in OA. A previous study indicated that duloxetine, an antidepressant, was effective to alleviate not only MDD but also pain in OA patients (8). Therefore, early identification of MDD may be crucial for improving the outcomes of patients with OA.

Although the coexistence of the chronic pain and MDD can tend to further aggravate the severity of both disorders (9–11), the underlying association between them is unclear. A review revealed that the common neuroplasticity mechanism changes may be an important route for the occurrence and development of the chronic pain and MDD (12). The neurotrophin hypothesis indicates that numerous neurotrophins are responsible for controlling the neuronal plasticity (13), as a result, the detection of blood neurotrophins may be a potential approach to identify MDD in OA. Recently, Shi et al. performed a comprehensive meta-analysis on the blood neurotrophins for the clinical application of MDD and revealed that significantly reduced brain-derived neurotrophic factor (BDNF) and significantly increased insulin-like growth factor-1 (IGF-1), vascular endothelial growth factor (VEGF), and S100B protein may be potential diagnostic biomarkers for MDD (14). Meanwhile, Stefani et al. also found that abnormal serum levels of BDNF and S100B were associated to the chronic pain (15). Thus, it is essential to assess the clinical value of these neurotrophins in OA.

The present study aimed to investigate whether the BDNF, VEGF, S100B, and IGF-1 in serum levels can be helpful to identify MDD in knee OA patients and to evaluate the potential relationship of these neurotrophins with depression and pain assessments. Furthermore, a 3-year follow-up was conducted in the present study, and the dynamic characteristics of these serum indicators and the symptoms of depression and pain in knee OA patients were also assessed.

## Materials and methods

### Participants

A total of 215 participants with knee OA presenting to the outpatient department were recruited from the Xiangyang Central Hospital, and among them, 134 patients with a history of depression (defined as the MDD group) and 81 ones without a depressive behavior (defined as the control group). The present study was approved by the Ethics Committee of Xiangyang Central Hospital (approval No. XYCH2017-023), and all participants provided signed informed consent.

A diagnosis of knee OA was made by an orthopedic surgeon based on the American College of Rheumatology clinical criteria (16). The inclusion criteria included (1) between 45 and 80 years of age; (2) knee pain of duration ≥ 6 months; (3) ≥2 scores of the Kellgren and Lawrence osteoarthritis scale (17); (4) being able to stand up independently from a chair and lay prone; and (5) body mass index ≤ 30. Furthermore, MDD was diagnosed according to the Diagnostic and Statistical Manual of Mental Disorders, Fourth Edition. MDD patients satisfied the 17-item Hamilton Depression Scale (HAMD-17) score of ≥8 and treatment-naïve, and each patient had no family history of a mental disorder. For all participants, the following exclusion criteria were applied: (1) any neurological illnesses; (2) lower limb fractures, post traumatic knee osteoarthritis, or head trauma; (3) total knee arthroplasty; (4) unexplained radiating leg pain; (5) secondary mental disorders; (6) alcohol or drug abuse; (7) active tumors; (8) history of significant physical disorder (e.g., endocrine disease, autoimmune disease or liver or kidney dysfunction); and (9) pregnancy and breastfeeding.

A 3-year observational follow-up was conducted for all participants of the control group, and among 81 OA participants, 39 participants transformed into depression (defined as the trans-MDD group) and others kept non-depression (defined as the non-MDD group).

Additionally, no specific intervention (i.e., surgical treatment) was conducted for each OA participant, and non-surgical treatment that included non-pharmacological and pharmacological treatment were recorded (please see Table 1). Non-pharmacological treatment consisted of education, weight management, and strength training exercises, and pharmacological treatment comprised paracetamol and oral non-steroidal anti-inflammatory drugs. As an observational study, no new treatment was assigned to participants during the follow-up, and for each OA participants, the treatment type was not changed until the end of follow-up.

**TABLE 1:** The clinical features and serum levels of neurotrophins in knee OA patients at baseline and after 3-year follow-up.

Baseline	MDD (*n* = 134)	Control (*n* = 81)	*P*-value
Age (years)	62.60 ± 11.16	61.50 ± 9.37	0.699^b^
Sex (Male/Female)	60/74	46/35	0.058^c^
BMI (years)	22.82 ± 3.29	22.60 ± 3.73	0.642^a^
Pain of duration (months)	48.67 ± 36.94	45.37 ± 35.30	0.544^b^
HAMD-17 scores	29.84 ± 9.67	4.09 ± 1.93	< 0.001^b^
SDS scores	52.66 ± 19.27	34.32 ± 8.09	< 0.001^b^
VAS scores	5.22 ± 2.50	2.46 ± 1.10	< 0.001^b^
BDNF levels (ng/ml)	18.25 ± 5.45	24.15 ± 5.19	< 0.001^a^
VEGF levels (pg/ml)	159.54 ± 46.44	134.06 ± 48.14	< 0.001^b^
S100B levels (pg/ml)	19.37 ± 5.78	15.52 ± 5.04	< 0.001^a^
IGF-1 levels (ng/ml)	96.72 ± 27.47	103.61 ± 31.60	0.105^a^
Treatment type (Non-pharmacological/Pharmacological)	101/33	67/14	0.137^c^

**Three-year follow-up**	**Non-MDD (*n* = 42)**	**Trans-MDD (*n* = 39)**	***P*-value**

HAMD-17 scores (baseline)	4.02 ± 2.07	4.15 ± 1.80	0.851^b^
HAMD-17 scores (3-year)	5.81 ± 2.57	15.95 ± 6.96	< 0.001^b^
SDS scores (baseline)	34.74 ± 9.50	33.87 ± 6.33	0.633^a^
SDS scores (3-year)	36.90 ± 9.80	40.92 ± 7.15	0.039^a^
VAS scores (baseline)	2.64 ± 0.98	2.26 ± 1.19	0.142^b^
VAS scores (3-year)	3.40 ± 2.00	4.49 ± 2.05	0.030^b^
BDNF levels (baseline) (ng/ml)	25.68 ± 5.53	22.49 ± 4.28	0.005^a^
BDNF levels (3-year) (ng/ml)	24.30 ± 16.20	16.20 ± 5.34	< 0.001^a^
VEGF levels (baseline) (pg/ml)	138.40 ± 51.50	129.40 ± 44.42	0.210^b^
VEGF levels (3-year) (pg/ml)	154.68 ± 46.31	138.83 ± 35.55	0.090^a^
S100B levels (baseline) (pg/ml)	13.28 ± 3.62	17.94 ± 5.28	< 0.001^b^
S100B levels (3-year) (pg/ml)	14.82 ± 3.95	20.43 ± 5.18	< 0.001^a^
Treatment type (Non-pharmacological/Pharmacological)	30/6	37/8	0.568^c^

OA, osteoarthritis; MDD, major depressive disorder; BMI, body mass index; HAMD-17, 17-item Hamilton Depression Scale; SDS, Self-Rating Depression Scale; VAS, Visual Analogue Scale; BDNF, brain-derived neurotrophic factor; VEGF, vascular endothelial growth factor; IGF-1, insulin-like growth factor-1.

^a^The analysis was performed using independent-sample *t*-test.

^b^The analysis was performed using Mann–Whitney test.

^c^The analysis was performed using chi-squared test.

### Assessment tools

The HAMD-17 as a primary test tool, was used to assess depressive symptoms, and the Self-Rating Depression Scale (SDS) were also used as the second test tool to assess the severity of depression. Meanwhile, the Visual Analogue Scale (VAS) was used to measure pain intensity in knee pain. The higher the score of these scales, the more severe of symptoms.

### Sample collection

Venous blood was collected from each OA participant after overnight fasting into a vacutainer tube (without anticoagulant). Within 30 min of collection, the coagulated blood was centrifuged at 3500 rpm for 10 min at 4°C to obtain serum. The serum samples were aliquoted and stored at −80°C. Blood was obtained from the 81 OA participant in the control group at baseline and the end of 3-year observational follow-up, respectively. For participants in the MDD group, samples were collected once at baseline.

### Enzyme-linked immunosorbent assay analyses

The concentrations of serum neurotrophins (BDNF, VEGF, S100B, and IGF-1) were measured using the commercial ELISA kits (R&D Systems, Minneapolis, MN, USA; BDNF: catalog No.DBD000; VEGF: catalog No. DVE00; S100B: catalog No. DY1820-05; IGF-1: catalog No. DG100B) in accordance with the manufacturer’s protocols. The concentration in each plate was determined using a microplate reader (Thermo Scientific™, Shanghai, China). Each sample was measured in triplicate, and the inter- and intra-assay coefficients of variation were <4%.

### Statistical analysis

Statistical analyses were performed using SPSS version 16.0 (SPSS, Inc., Chicago, IL, USA). The normal distribution of the data was assessed using the Kolmogorov-Smirnov test, and the Levene’s homogeneity of variance test was also performed. Categorical variable (i.e., sex) was analyzed using the chi-squared test, and continuous variables (i.e., age, pain of duration, body mass index [BMI], emotion and pain assessments, and serum neurotrophins’ levels) were analyzed using the independent-sample *t*-test or Mann–Whitney test. The paired *t*-test was used to determine the difference of variables before and after follow-up. Partial correlation analysis was used to determine a possible association of emotion or pain assessments with serum neurotrophins’ levels, controlling age, sex, pain of duration, BMI, and treatment types. Notably, the change value of serum neurotrophins level was calculated with “Δ = baseline − the end of follow-up,” and the change rate of depression or pain assessment was calculated with “%Δ = (baseline − the end of follow-up)/baseline.” *P* < 0.05 was considered as statistical significance (two tailed).

As described in our previous study (18), the mediation analysis was utilized to determine whether serum neurotrophins could mediate the association between depressive behaviors and knee pain, which is based on a standard three-variable mediation model (19). Covariates were age, sex, pain of duration, BMI, and treatment types.

The area under the curve (AUC) of the receiver operating characteristic curve (ROC) was calculated to determine the distinguishing ability of serum neurotrophins which exhibited significant difference between two groups. The Youden index (20) was used to obtain optimal values of sensitivity and specificity. In addition, the binary logistic regression (21) (dependent variable: MDD/control; independent variable: serum neurotrophins) was used to calculate a predicted value of each patient that can be regarded as the combined indicator based on these serum neurotrophins.

## Results

### Characteristic of participants at baseline

In the Table 1, 134 OA participants showed significantly increased the HAMD-17 and SDS scores when compared with the other 81 OA participants. Therefore, these OA participants were divided into the MDD group (*N* = 134) and the control group (*N* = 81).

The MDD group showed significantly higher VAS scores and serum levels of VEGF and S100B than the control group (Table 1). Meanwhile, the serum levels of BDNF was significantly reduced in the MDD group as compared to the control group (Table 1). However, the serum IGF-1 levels had no significant difference between MDD and control groups (Table 1). Furthermore, the clinical characteristics (e.g., age, sex, BMI, pain of duration, and treatment types) were non-significantly different between two groups (Table 1).

### Relationship of serum neurotrophins with emotion and pain assessments in the major depression disorder group at baseline

**FIGURE 1:**
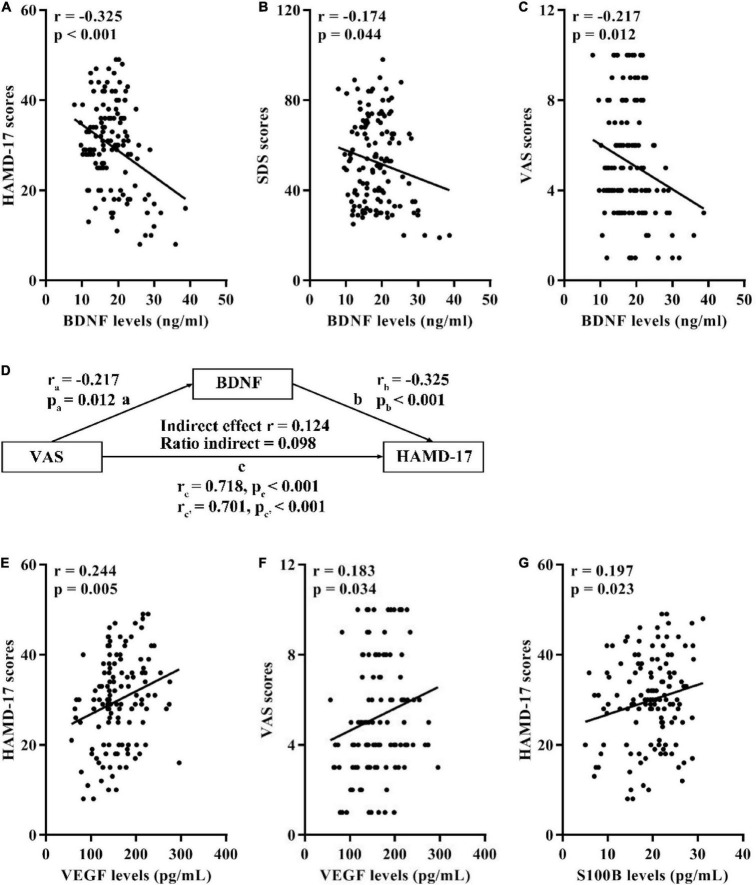
Associations of serum BDNF, VEGF, and S100B with the HAMD-17, SDS, and VAS scores in 134 OA participants with MDD. **(A)** Correlation between BDNF levels and the HAMD-17 scores. **(B)** Correlation between BDNF levels and the SDS scores. **(C)** Correlation between BDNF levels and the VAS scores. **(D)** Mediation effects of serum BDNF on the association between the HAMD-17 and VAS scores. **(E)** Correlation between VEGF levels and the HAMD-17 scores. **(F)** Correlation between VEGF levels and the VAS scores. **(G)** Correlation between S100B levels and the HAMD-17 scores. BDNF, brain-derived neurotrophic factor; VEGF, vascular endothelial growth factor; HAMD-17, 17-item Hamilton Depression Scale; SDS, Self-Rating Depression Scale; VAS, Visual Analogue Scale; OA, osteoarthritis; MDD, major depressive disorder.

Correlation analyses in 134 OA participants with MDD demonstrated that there were negative correlations between serum BDNF levels and the HAMD-17, SDS, and VAS scores (Figures 1A–C). Besides, mediation analysis indicated that serum levels of BDNF significantly mediated the effect of the VAS scores on the HAMD-17 scores (Figure 1D) where covariates were controlled for age, sex, pain of duration, BMI, and treatment types.

Furthermore, the serum levels of VEGF was positively correlated with the HAMD-17 and VAS scores (Figures 1E,F) in MDD patients. Moreover, there was a positive correlation between serum S100B levels and the HAMD-17 scores (Figure 1G).

### Characteristic of osteoarthritis participants in the control group after 3-year follow-up

At the end of 3-year follow-up, 39 OA participants showed depressive behaviors, who were assigned to the trans-MDD group, and 42 OA participants as the non-MDD group, didn’t display any depressive disorders.

As shown in Table 1, baseline HAMD-17, SDS, and VAS scores, baseline serum VEGF levels, and treatment types were not significantly different between the trans-MDD and non-MDD groups. Meanwhile, baseline serum levels of BDNF and S100B had significant difference between two groups (Table 1).

After the follow-up, 39 OA participants in the trans-MDD group showed significantly lower serum BDNF levels and higher serum S100B levels than 42 ones in the non-MDD group (Table 1). However, serum levels of VEGF after the follow-up exhibited no significant difference between two groups (Table 1).

**FIGURE 2:**
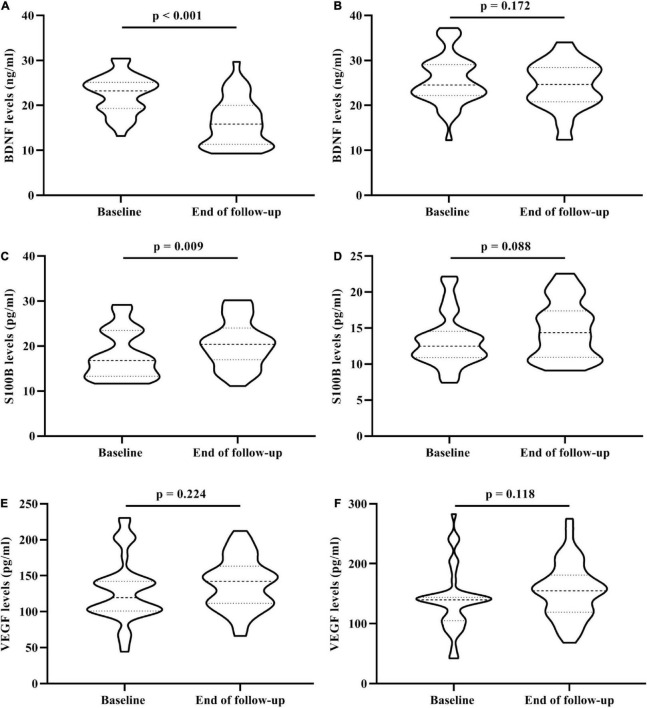
The change of serum BDNF, VEGF, and S100B levels in 81 OA participants who underwent the 3-year follow-up. **(A)** The change of serum BDNF levels between baseline and the end of follow-up in trans-MDD group. **(B)** The change of serum BDNF levels between baseline and the end of follow-up in non-MDD group. **(C)** The change of serum S100B levels between baseline and the end of follow-up in trans-MDD group. **(D)** The change of serum S100B levels between baseline and the end of follow-up in non-MDD group. **(E)** The change of serum VEGF levels between baseline and the end of follow-up in trans-MDD group. **(F)** The change of serum VEGF levels between baseline and the end of follow-up in non-MDD group. BDNF, brain-derived neurotrophic factor; VEGF, vascular endothelial growth factor; OA, osteoarthritis; MDD, major depressive disorder.

In addition, baseline serum BDNF and S100B levels changed significantly after the follow-up in the trans-MDD group, however, there was no significant difference in in the non-MDD group (Figures 2A–D). For serum VEGF levels, neither the trans-MDD group nor the non-MDD group had significant difference between baseline and the end of follow-up (Figures 2E,F).

### Correlation analysis in the trans-major depression disorder group after the 3-year follow-up

**FIGURE 3:**
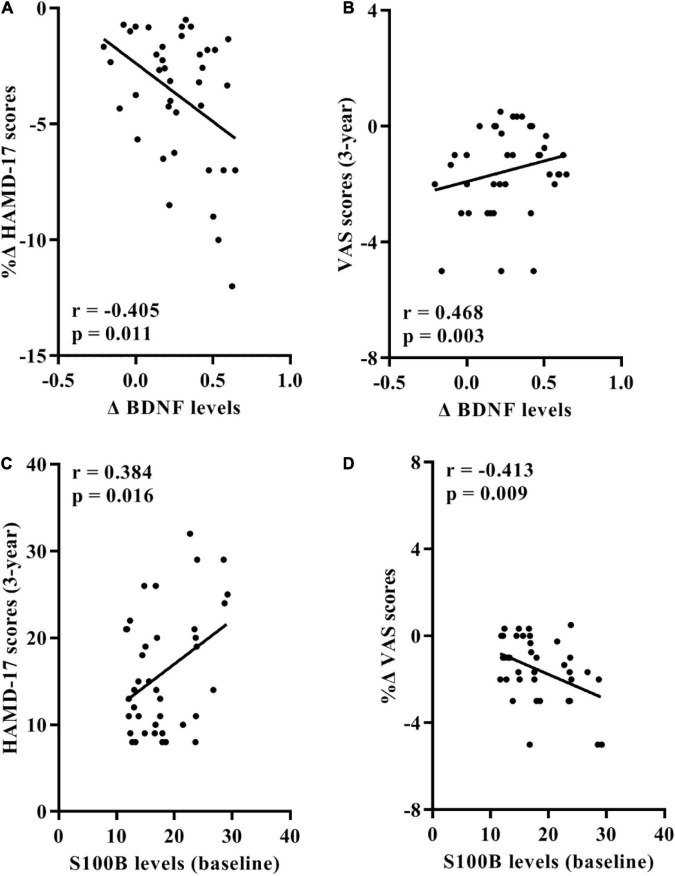
Associations between serum BDNF/S100B levels and the HAMD-24 and VAS scores in 39 participants of trans-MDD group after the 3-year follow-up. **(A)** Correlation between rates of change of HAMD-17 scores and the change value of BDNF levels. **(B)** Correlation between the change value of BDNF levels and the VAS scores at the conclusion of the 3-year follow-up. **(C)** Correlation between the HAMD-17 scores at the end of the 3-year follow-up and serum S100B levels at baseline. **(D)** Correlation between rates of change of VAS scores and serum S100B levels at baseline. BDNF, brain-derived neurotrophic factor; HAMD-17, 17-item Hamilton Depression Scale; VAS, Visual Analogue Scale; OA, osteoarthritis; MDD, major depressive disorder.

At the conclusion of the 3-year follow-up in the trans-MDD group, a significant negative correlation between the change of serum BDNF levels and rate of change of HAMD-17 scores was observed (Figure 3A), with the change of serum BDNF levels positively associated with the VAS scores (Figure 3B).

Furthermore, baseline serum S100B levels positively correlated with the HAMD-17 scores at the end of follow-up and negatively correlated with the change rate of VAS scores (Figures 3C,D).

### Receiver operating characteristic curve analyses

**FIGURE 4:**
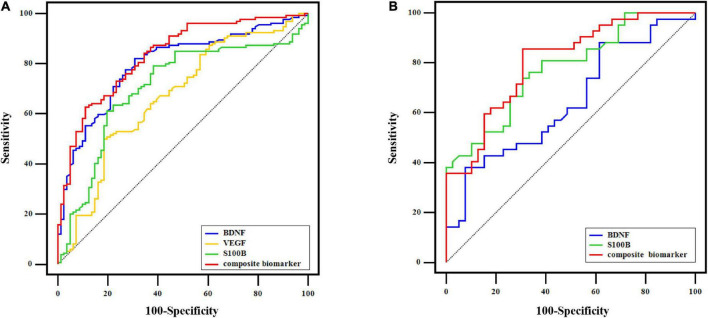
ROC curve analysis. **(A)** In 215 OA participants, ROC curves of BDNF, VEGF, S100B, and the composite biomarker of their combination for distinguishing MDD from control at baseline. **(B)** ROC curves of baseline BDNF, baseline S100B, and the combination of the two indicators to distinguish OA participants who transformed into MDD from those with non-MDD after the follow-up.

Figure 4A displays the AUC value of BDNF, VEGF, and S100B at 0.799 (95% CI: 0.739∼0.850), 0.668 (CI: 0.600∼0.730), and 0.706 (95% CI: 0.640∼0.766), respectively, for distinguishing MDD patients from controls in all OA participants. However, the combination of BDNF, VEGF, and S100B provided greater diagnostic power with an AUC value of 0.834 (95% CI: 0.778∼0.881), corresponding to a specificity of 88.89% and a sensitivity of 62.69% for identifying MDD in OA participants (Figure 4A).

As shown in Figure 4B, the combined indicator of baseline BDNF and S100B was able to differentiate OA participants in the trans-MDD group from the non-MDD group with the AUC value of 0.806 (95% CI: 0.703∼0.885; specificity: 69.23%; sensitivity: 85.71%), which was better than single baseline BDNF (AUC = 0.644; 95% CI: 0.530∼0.747) or baseline S100B (AUC = 0.777; 95% CI: 0.670∼0.862).

## Discussion

The present study firstly determined the feature of serum neurotrophins in knee OA patients with MDD and the potential association of serum neurotrophins with depression and pain symptoms. The main findings in the present study are as follow: (1) in comparison with OA participants without depressive behaviors, a significant decrease in serum BDNF and significant increase in serum VEGF and S100B and VAS scores in OA participants with MDD were observed; (2) in OA participants, there were significant correlations between serum levels of BDNF, VEGF, and S100B and depression and pain assessment scores in MDD patients; (3) particularly, a mediation of the association was found between the VAS scores and the HAMD-17 scores through the BDNF as mediator in OA participants with MDD; (4) significantly lower baseline BDNF levels and higher baseline S100B levels were detected in OA participants who transforming to MDD after a 3-year follow-up when compared with those who showed normal behaviors; (5) at the end of follow-up, significant associations of serum BDNF and S100B with the performance of depression and pain were found in the trans-MDD group; (6) in OA participants, the composite indicator of BDNF, VEGF, and S100B had optimal power to differentiate MDD patients from controls, and the combination of BDNF and S100B could effectively predict the transformation of MDD after the 3-year follow-up. Taken together, serum BDNF, VEGF, and S100B may be a potential diagnostic biomarker of MDD in knee OA, with serum BDNF and S100B in particular being able to predict the occurrence of MDD in the development of OA.

Several previous studies had found that in various body fluids (e.g., plasma, synovia), OA patients showed significant higher BDNF levels, and BDNF/TrkB signaling contributes to developing chronic OA pain (22–24). As a driving force behind neuroplasticity, BDNF-related signal pathway not only influences the sensitization of pain pathways but also restrains the negative effect of MDD (25, 26). In the present study, significantly increased BDNF in serum levels were found in OA participants with MDD, and OA participants who transformed into MDD after the follow-up also showed significantly increased serum BDNF levels at baseline and the end of follow-up, which suggested that serum BDNF may be used as a potential diagnostic and predictive biomarker to identify MDD in OA. Furthermore, in OA participants with MDD, the negative correlation between BDNF levels and the HAMD-17, SDS, and VAS scores suggests that the decrease in serum levels of BDNF may reflect the severity of depression and pain. Interestingly, a mediator model further found that pain can affect a depressive state through a change in serum BDNF levels, strongly indicating that BDNF may play an important role in mediating the association of the pain with MDD. Recently, Dimmek et al. detected that in OA-related chronic widespread pain patients, reduced serum BDNF levels may induce the anxiety and depression *via* affecting the function of immune cells (27), which supported the present findings on relationships of BDNF with depression and pain. Additionally, previous studies showed serum/plasma BDNF levels can elevated in OA patients after the continuous non-surgical treatment, with improving the pain symptom (28, 29), which suggested that BDNF levels can be affected by the severity of OA. Similarly, the present study observed that in the development of OA, lower serum BDNF levels reflected the more serious of pain and predicted the high risk of MDD. Although low serum BDNF levels were also found and antidepressants could increase BDNF levels in depressed patients (30), a meta-analysis provided an overall increased trend of BDNF in MDD patients and higher serum BDNF levels may play an important role in the pathophysiology of MDD (14). Consequently, serum BDNF as a valuable indicator may contribute to early identifying MDD in OA patients.

S100B is a calcium-binding protein produced predominantly by astrocytes, and is able to enhance neuroplasticity (31). Previous studies showed that S100B levels were significantly increased in serum, synovial fluid, and knee synovial tissue of OA patients and had an effect on the repair of cartilage damage (15, 32, 33). Meanwhile, S100B was also involved in the pathology of MDD and showed significant increase in peripheral blood and autopsy brain tissues individuals with MDD (14, 34, 35). The present study displayed that in OA participants, patient with MDD showed significantly elevated serum S100B levels and positive correlation between S100B levels and the HAMD-17 scores. Importantly, our study also found that increased serum S100B levels in OA patients would aggravate the chronic pain and lead to the occurrence of MDD with the development of OA. Although the role and mechanism of S100B in OA is unclear, the clinically diagnostic and predictive value of S100B for MDD had been determined in OA, which is helpful to conduct the early antidepressive strategies for OA.

Vascular endothelial growth factor is expressed in articular cartilage and increases in serum levels have been associated with the progression of OA (36, 37). Previous studies showed that inhibition of VEGF-related signaling pathway (e.g., PIM2/VEGF signaling, VEGF-A/VEGFR2 signaling) can attenuate the early development of OA, and several therapies targeting VEGF and its receptors had been investigated for the treatment of OA (38–40). Furthermore, VEGF is a vascular permeability factor, and when VEGF levels are pathologically elevated, blood brain barrier integrity may be impaired, with affecting central nervous system homeostasis, which may lead to the dysfunction of brain and the emergence of MDD (41, 42). In the present study, OA participants with MDD showed significantly higher serum VEGF levels than those with non-MDD, and the significantly positive associations of VEGF levels with the HAMD-17 and VAS scores were also observed in OA participants with MDD, which suggested that VEGF may be an important indicator to reflect the severe of OA, especially psychosomatic symptoms. Although in OA participants, the abnormal change of serum VEGF levels was not observed between trans-MDD and non-MDD groups after the 3-year follow-up, VEGF is still a vital target for the treatment of OA accompanying MDD.

Some previous studies reported that higher serum IGF-1 concentration presented a high risk of knee OA (43, 44), however, lower serum IGF-1 levels were also found in other studies (45, 46). Although IGF-1 is involved in the proliferation and differentiation of chondrocyte and the synthesis of collagen and glycosaminoglycan (45, 47, 48), the role of IGF-1 in OA pathogenesis is unknown. In the present study, no difference in serum IGF-1 levels between MDD and control groups may be associated with the inconsistent changes of IGF-1 levels in OA patients (43–46). Further investigation is necessary to evaluate the clinical value of IGF-1 in OA with MDD.

In the present study, our findings provide evidence for the diagnosis of MDD in OA patients using serum neurotrophins. Compared with the single indicator, a panel of BDNF, VEGF, and S100B is recommended for optimal differential diagnosis of MDD. In addition, we also found that the collective contribution of BDNF and S100B is an optimal combined biomarker for predicting the high risk of MDD in OA patients.

However, several limitations in the present study need be considered. Firstly, OA participants in the present study did not restricted the treatment type, and during the follow-up, individual treatment of each OA patient was also uncontrolled. Although confounding factors were adjusted in the data analysis, different treatment types may have an effect on the serum neurotrophins levels. Treatment-free OA patients will be focused on in the future study. Secondly, magnetic resonance imaging is the important means for whole-joint evaluation in OA study and brain function evaluation in MDD research, however, analysis of imaging data was lacking in the present study. We will further improve the imaging analysis in the subsequent study. Thirdly, the larger sample size is essential to further assess serum neurotrophins in OA patients with different degrees of MDD. Lastly, commercially available BDNF ELISA kit can recognize both BDNF (mature) and its precursor proBDNF in the human blood, thus, the present levels of BDNF are the total BDNF levels including BDNF (mature) and proBDNF (49, 50).

## Conclusion

The present study demonstrated that OA patients with MDD showed significantly changed serum BDNF, VEGF, and S100B and significant associations of these neurotrophins with depression and pain assessments, which suggested that these indicators may be potential biomarkers to identify MDD in OA patients. Furthermore, findings of the follow-up study further found that serum BDNF and S100B were potential biomarkers to predict the risk of MDD for OA.

## Data availability statement

The original contributions presented in this study are included in the article/supplementary material, further inquiries can be directed to the corresponding authors.

## Ethics statement

The studies involving human participants were reviewed and approved by the Ethics Committee of Xiangyang Central Hospital. The patients/participants provided their written informed consent to participate in this study. Written informed consent was obtained from the individual(s) for the publication of any potentially identifiable images or data included in this article.

## Author contributions

XL and GC designed the study, contributed to the discussion, and revised the manuscript. PZ drafted the manuscript. BW, YZ, and ZW recruited the participants, completed the assessments, and collected the blood samples. YX and JS conducted the ELISA research. PZ and CL contributed to the data analyses and discussion. All authors contributed to the article and approved the submitted version.
